# Delivery of RNA and Its Intracellular Translation into Protein Mediated by SDS-CTAB Vesicles: Potential Use in Nanobiotechnology

**DOI:** 10.1155/2013/734596

**Published:** 2013-05-29

**Authors:** Laura Russo, Valerio Berardi, Franco Tardani, Camillo La Mesa, Gianfranco Risuleo

**Affiliations:** ^1^Dipartimento di Biologia e Biotecnologie “Charles Darwin,” Sapienza Università di Roma, Piazzale Aldo Moro 5, 00185 Roma, Italy; ^2^SUNY Downstate Medical Center, 450 Clarkson Avenue, Brooklyn, NY 11203, USA; ^3^Dipartimento di Chimica “Stanislao Cannizzaro,” Sapienza Università di Roma, Piazzale Aldo Moro 5, 00185 Roma, Italy

## Abstract

Catanionic vesicles are supramolecular aggregates spontaneously forming in water by electrostatic attraction between two surfactants mixed in nonstoichiometric ratios. The outer surface charges allow adsorption to the biomembrane by electrostatic interactions. The lipoplex thus obtained penetrates the cell by endocytosis or membrane fusion. We examined the possible cytotoxic effects and evaluated the transfection efficiency of one vesicle type as compared to known commercial carriers. We show that the individual components of two different vesicles types, CTAB (cetyltrimethylammonium bromide) and DDAB (didodecyldimethylammonium bromide) are detrimental for cell survival. We also assayed the cytotoxicity of SDS-DDAB vesicles and showed dose and time dependency, with the DDAB component being *per se* extremely cytotoxic. The transfection efficiency of exogenous RNA mediated by SDS-CTAB increases if vesicles assemble in the presence of the reporter RNA; finally, freezing abrogates the transfection ability. The results of our experimental strategy suggest that catanionic vesicles may be adopted in gene therapy and control of antiproliferative diseases.

## 1. Introduction

Catanionic vesicles are supramolecular aggregates formed by mixing in non-stoichiometric ratios cationic and anionic surfactant species [1]. Surfactants of opposite charge tend to aggregate in polar solvents, such as water. The electrostatic interactions between the polar heads and hydrophobic tails favor the formation of self-assembled and organized supramolecular structures [2]. The occurrence of this phenomenon depends upon the so-called “critical micellar concentration” (CMC) which represents the concentration where the surfactants in solution aggregate to form spontaneously micelles with different morphologies. In addition, the diverse shapes depend on the geometry of the individual surfactant molecule. The relationship between molecular geometry of the surfactant and the morphology of the self-organized structures can be determined by the packing parameter *P*, originally introduced by Israelachvili and coworkers [3]. This parameter is defined as the ratio between the volume of the hydrophobic tract (*V*) and the area of the polar head (*a*) multiplied by the length of the chain (*L*) being part of the same surfactant (*P* = *V*/*aL*). The modulus of *P* is suggestive of the type of structure/shape that surfactants tend to assume upon aggregation. The formation of vesicles is, therefore, possible when the packing parameter reaches an optimal value thus leading to the formation of a double layer [4]. 

Previous work from our laboratory, where the phase diagram was presented, indicated that to form quasispherical vesicles, it is necessary to mix the surfactants at nonequimolar concentrations since at stoichiometric ratio the two components precipitate [1]. 

Concerning the potential use of these complexes in biotechnological and biomedical applications, it should be pointed out that the interaction of vesicles with other molecules, such as DNA, RNA, or other biopolymers, results in the formation of the above mentioned lipoplex. This represents a potential tool to deliver genetic material across the cell membrane via plasma-membrane fusion and/or endocytosis. As a matter of fact, previous work from our laboratory showed that it is possible to form complexes between DNA and vesicles with an excess positive charge at the surface, which could be potentially delivered within the cell [5, 6]. However, vesicles may show a cytotoxic effect, which is directly related to time of exposure and dose of administration. The literature focused on this specific aspect is not very abundant [7, 8], even though recent work from our laboratory showed that tumor cells exhibit a higher sensitivity to treatment with SDS-CTAB vesicles as compared to normal mouse fibroblasts [9]. However, in spite of their cytotoxicity, it is possible to adjust the experimental conditions such as time and dose of treatment to allow a successful vesicle-mediated transfection with subsequent expression of exogenous genetic material [9–11]. In addition, pilot studies indicate that catanionic vesicles may be utilized in anticancer therapy [11]. 

In the present work, we report on the cytotoxic action of both the individual surfactants and vesicles formed by the same ones, on HEK-293 cultured cells. In addition, we quantitatively evaluated the transfection, mediated by SDS-CTAB vesicles, of an exogenous RNA and measured the level of translation of the reporter protein. Strictly speaking, in fact, one should talk in terms of messenger RNA translation rather than gene expression. The data discussed here clearly indicate that after transfection, the nucleic acid is translated into protein with the correct structure and at abundant level. The novelty of the data discussed here consists of the fact that naked RNA, a very vulnerable biomacromolecule, is protected by the interaction with vesicles. Finally, these data add further evidence that vesicles may find a use in biotechnology and gene therapy.

## 2. Experimental Section

### 2.1. Vesicle Preparation and Characterization

Vesicles were prepared and characterized as previously published and extensively discussed [5, 6, 9]. Briefly, the micellar solutions of SDS and DDAB were mixed in water, The individual concentrations for each surfactant were 0,02133 g (SDS) and 0,01607 g (CTAB or DDAB) in 10.00 g of H_2_O. Purification was made by dissolving the individual species in hot ethyl alcohol, under stirring with subsequent precipitation and filtering the surfactant solution by cold acetone. The salts were recovered and vacuum-dried at 70°C. Their purity was determined by conductivity methods in water at 25.0°C. Mixtures of the single species were prepared in conductivity water (*χ* close to 1 ∗ 10^−7^ Ω^−1^ cm^−1^, at room temperature) and heated above the respective Krafft points of each surfactant [12]. Thereafter, the solutions were mixed together. The dynamic light scattering (DLS) unit determining vesicle size is a Malvern Zeta Nanosizer, working at 632.8 nm in back scattering mode.

### 2.2. Cell Cultures and *In Vivo* Translation

Cells were maintained according to standard cell culture protocols. Transfection experiments *in vivo* were done in a Human Embryonal Kidney cell line (HEK-293). In general, 1 × 10^5^ cells per well were seeded on 12 well polylysine-treated plates without antibiotics. Cells were allowed to reach at least 80% confluence prior to transfection. We used Plasmid pOT.CAT-A98 to generate CAT-A98 mRNAs. The RNAs used for transfection were transcribed *in vitro* using a commercial kit (RiboMax) and were phenol-chloroform-extracted (5 : 1 v/v at pH 4.7), ethanol-precipitated, and further purified on Sephadex G-50 or G-25 columns (GE Healthcare); CAT-A98 mRNAs were 5′-capped and polyadenylated. As control, CAT-A98 mRNAs were transfected using Lipofectamine 2000 (Invitrogen) in Opti-MEM culture medium (Invitrogen). Briefly, transfections were performed with RNA and Lipofectamine at a ratio of 1 : 2.5 as previously published [13]. CAT was transfected at different molar concentrations (as indicated in the captions of the figures). Where appropriate, CAT mRNA was added before the formation of vesicles, or alternatively to preformed ones. Cells were exposed to the RNA/transfection mixture for 4 hours. Subsequently, this was replaced with fresh, serum-containing medium, and incubation was continued for 3 hours. Cells were collected, lysed, and probed for CAT protein using a CAT-ELISA Kit (Roche). ELISA data were normalized to total protein concentrations of cells assessed by the Bradford assay [14].

### 2.3. Evaluation of Cell Viability

Cell viability was evaluated by the colorimetric Mosmann assay [15] on both HEK-293 and 3T6 cells (see also legends of the figures). This quantitative method has been extensively illustrated in previous works, for instance see [9]. In any case, absorbance values measured at 570 nm can be directly converted into number of vital cells. 

### 2.4. Statistics

All experiments and measurements were independently replicated 3 times. Statistical analysis was done by one-way ANOVA test [12] followed by comparative LSDs (Least Significant Differences) test. Results were considered significant when *P* < 0.05.

## 3. Results and Discussion

### 3.1. Estimates of Vesicle Size and Charge Density

**Figure 1:**
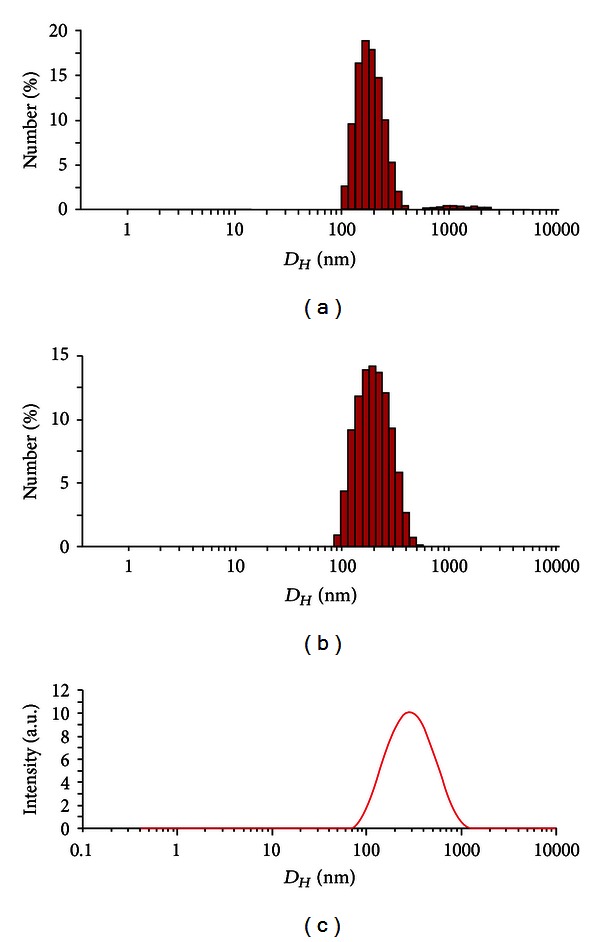
Estimation of the vesicle size by DLS. Size distribution of the SDS-DDAB vesicle monitored prior (a) and after filtration (b). (c) Integration of the data reported in panel (b). Concentration of the vesicle was 3.8 mmol kg^−1^; the total surfactant concentration was 4.05 mmol kg^−1^. The pore size of the filter was 450 nm.

The vesicular mixtures were prepared as previously reported [1, 5, 6, 9]. The shelf life of these dispersions is up to four months long, and we could not observe any tendency to phase separation over a three-month period. The thermodynamic stability of the vesicles is controlled by several parameters such as the overlapping of curvature elasticity, bending energy, and electrostatic terms [16–19]. The surface charge density is governed by the partition of the two surfactants between the bilayer and the bulk and is therefore controlled by Gibbs energy of transfer for the two species [17, 18]. DLS gives relevant information on vesicle size and polydispersity. In our case, the average hydrodynamic value is centered on 300 nm (Figure 1(c)). A single population is evident with a unimodal distribution function, which is comparable to other vesicular dispersions of the catanionic family [9, 20–24]. The negligible peak observed in Figure 1(a) is attributable to a very small fraction of aggregated vesicles which, as a matter of fact, is eliminated by filtration (Figure 1(b)). 

### 3.2. Cytotoxicity of Individual Surfactants and Vesicles

**Figure 2:**
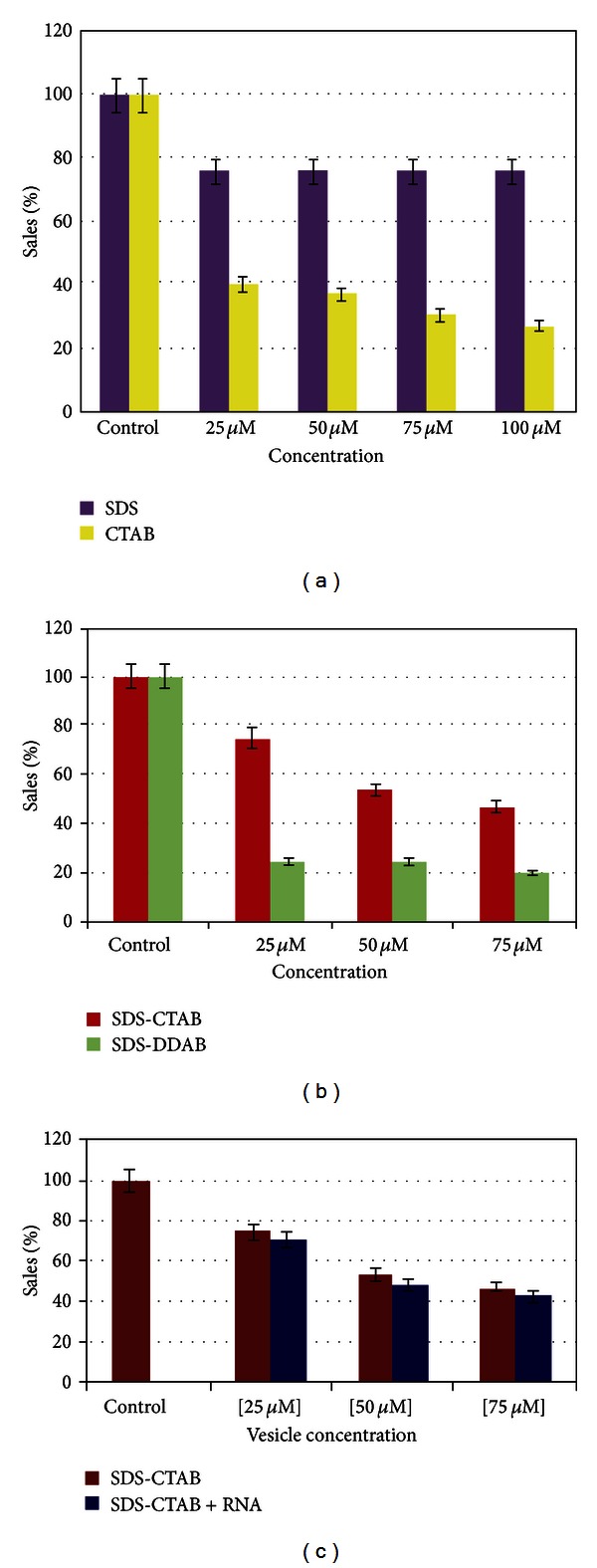
Effect of the separate surfactants and vesicles on the viability of HEK-293 cells. (a) Purple bars show the effect of SDS, while yellow bars show the toxicity of CTAB. (b) Compared cytotoxicity of SDS-CTAB (red bars) and SDS-DDAB (green bars). (c) Cytotoxicity of vesicle/RNA lipoplexes.

In this work, we compared the cytotoxic action of the individual surfactants SDS, CTAB, and DDAB on HEK-293 cells. Treatment time was four hours at increasing vesicle concentrations. The four-hour treatment time was chosen since this is required in the subsequent transfection experiments. In any case, it is worth noting that the CTAB component is more toxic than SDS (Figure 2(a)). Moreover, the mortality rate is directly proportional to the concentration for both surfactants. DDAB *per se* exhibited a dramatically higher toxicity than CTAB (not shown). 

In a second series of experiments, we have compared the toxic effects of two different vesicles, SDS-CTAB and SDS-DDAB, after four hours of exposure and in the same range of concentrations used in the previous experiments. As shown in Figure 2(b), the SDS-DDAB vesicles are far more toxic than the SDS-CTAB ones. This validates the former results obtained with the individual surfactants (Figure 2(a)) and suggests that the higher cytotoxic effect is mainly due to the DDAB component forming the vesicular aggregate. This is the rational for the use of SDA-CTAB vesicles in all subsequent experiments. Cytotoxicity of vesicles with bound RNA was also investigated (Figure 2(c)). Results show a slight increase in cell mortality which may be ascribed to the toxic effect of free RNA present in the mixture. 

### 3.3. RNase Protection Assay: Transfection of Chloramphenicol-Acetyltransferase Reporter mRNA

Chloramphenicol-Acetyltransferase (CAT) is a bacterial enzyme whose messenger RNA (mRNA) can be translated into active protein in eukaryotic cells. We transfected the CAT mRNA into HEK-293 cells using SDS-CTAB vesicles. The rationale of these experiments is that CAT is not normally present in higher cells; therefore, the detection of this enzyme is the diagnostic sign that the CAT mRNA has been successfully transferred across the plasma membrane by the vesicles and, subsequently, translated into protein within the cytoplasm matrix. We quantified the intracellular concentration of enzyme by the immunoenzymatic assay ELISA; finally, the level of CAT was normalized to the total protein content of the cells. 

**Figure 3:**
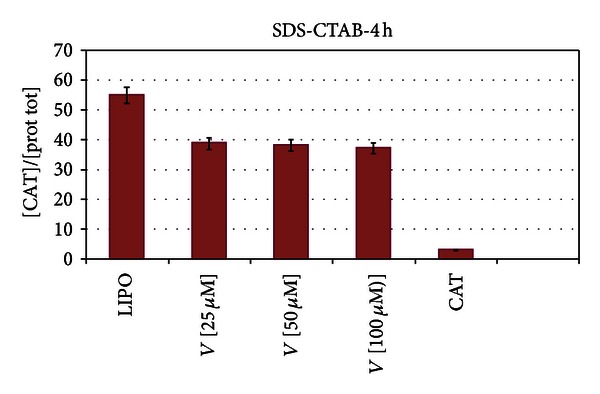
Transfection efficiency of mRNA-CAT. In this experiment, the RNA was added to preformed vesicles. The intracellular level of CAT is lower in the case of SDS-CTAB vesicles as compared to a commercial transfection system (Lipofectamine). See text for further details. LIPO (Lipofectamine); *V*[25 *μ*M] (25 *μ*M vesicle concentration); *V*[50 *μ*M] (50 *μ*M vesicle concentration); *V*[100 *μ*M] (100 *μ*M vesicle concentration); CAT (naked CAT mRNA).

**Figure 4:**
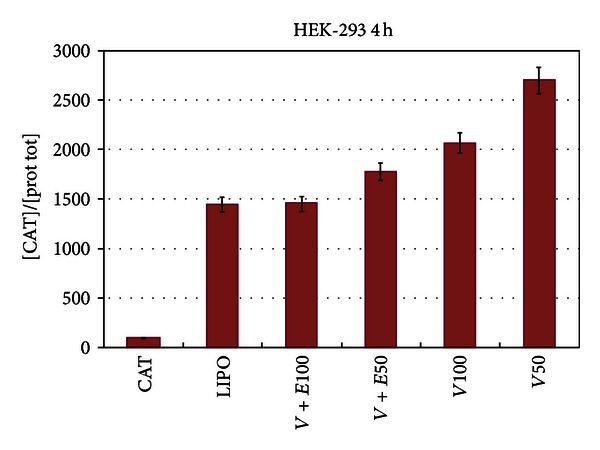
Transfection efficiency of mRNA CAT after treatment with RNase. In this experiment, the mRNA CAT was added to the surfactant mixture prior to the vesicle formation. This result strongly suggests that the RNA is internalized within the vesicle aqueous space and is thus protected by the nucleolytic attack. Panel (A): vesicles not treated with RNase (last two bars to the right) exhibit a higher efficiency than Lipofectamine. Panel (B): comparison between RNase untreated and treated vesicles. The numbers at the bottom of the bar indicate the vesicle concentration (*μ*M).

**Figure 5:**
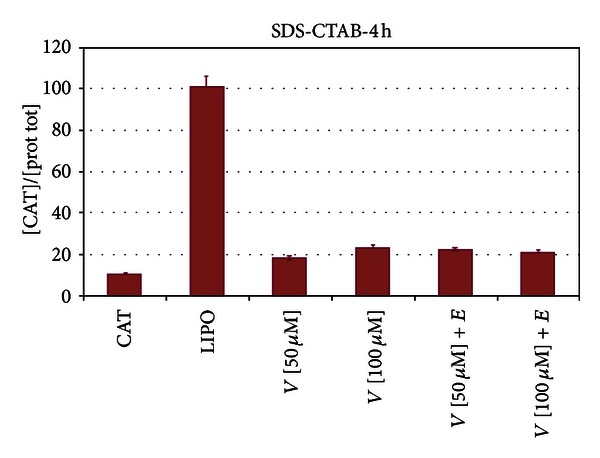
Effect of freezing on the transfection efficiency. Vesicles were formed in the presence of mRNA CAT and kept frozen at −20°C for 24 hours. After thawing, the aggregates were treated with RNase and transfected into HEK-293 cells. Data clearly indicate that the RNase treatment almost abolishes the translation of CAT mRNA into protein. LIPO (Lipofectamine); *V*[25 *μ*M] (25 *μ*M vesicle concentration); *V*[50 *μ*M] (50 *μ*M vesicle concentration); *V*[100 *μ*M] (100 *μ*M vesicle concentration); CAT (naked CAT mRNA).

We compared the efficiency of RNA intracellular delivery of our vesicles with Lipofectamine, a commercially available liposome transfection system. Transfection of naked CAT mRNA represented the negative control and, evidently, is almost totally hydrolyzed by the RNases normally present in the cytoplasm. As matter of fact, messenger RNA exists in a quasilinear molecular configuration which is easily hydrolyzed by the resident RNases. In the first experiments, we added the CAT mRNA to preformed vesicle. One can expect that in this case, the RNA is anchored via electrostatic interactions to the surface of the vesicles. The results of Figure 3 show that the immunoreaction between the CAT protein and anti-CAT antibody is, as expected, almost absent in the case of the transfection with naked CAT mRNA (bar to the right); this is consistent with the idea that the RNA is demolished by the RNases present in the cytoplasm and therefore becomes unavailable to be translated into protein. The transfection efficiency at the indicated concentrations of SDS-CTAB is indeed lower than the one exhibited by Lipofectamine (bar to the left) but is in any case quite satisfactory. One way to explain this lower efficiency is that the RNA located partly or totally on the external surface of the RNA is, again, attacked and inactivated by the cell RNases. Therefore, in the next experiments, we formed the vesicle in the presence of CAT mRNA: the assumption is that in this case the RNA would be included in the aqueous space internal to the vesicles. We treated these lipoplexes with RNase and subsequently transfected the cells as reported above. The results of Figure 4 show an improved transfecting performance of the vesicles, and Figure 4 demonstrates that in this case the RNA is protected by the nucleolytic attack. Actually, the efficiency increases with the concentration and becomes almost twice higher (at 100 *μ*M vesicles) with respect to Lipofectamine used as control (last bar to the left and first to the right, resp.). This strongly suggests that actually the RNA molecule is internalized and protected within the vesicle. Subsequently to transfection, the CAT mRNA is released in the cytoplasm where it can be translated into protein. In the last set of experiments discussed here, we probed the role of the storage temperature; as a matter of fact, previous evidence from our laboratory [1, 8] suggests that vesicles are quite stable in a temperature range of 15–25°C. To test the idea that freezing damages, or abolishes, the transfecting efficiency of the vesicles, we froze and kept them at a temperature of −25°C for about 24 hours. Data reported in Figure 5 clearly show that freezing almost abolishes the transfection capacity of the SDS-CTAB vesicles. The data of this ELISA assay demonstrate, in fact, that after freezing, the translation of the CAT mRNA drops almost to the same level as the one exhibited by the naked RNA (first bar to the left). Therefore, it is plausible to assume that freezing alters the supramolecular organization of the vesicles and consequently abrogates their activity of potential molecular biomachines for the delivery of bioactive polymers. As a matter of fact, from these data, we can infer that also other bio-macromolecules may be transferred inside the cells. One interesting aspect, yet to be investigated, is the mode of cell death. Previous evidence from our laboratory indicates that administration of vesicles to cultured cells causes apoptosis [9]. This is a multistep and very complex mode for a cell to die. Therefore, the elucidation of the key step in the process of cell death may help to set up the best experimental condition which, to minimal cell mortality, corresponds to an optimal delivery of the cargo macromolecule (or also small molecules) of biotechnological interest. Finally, a very important and original result shown in this work is that the interaction of CAT mRNA with the vesicles causes it to internalize within the supramolecular aggregate. To our knowledge, this is the first example of an mRNA being delivered within a cell and translated into a protein with a properly folded conformation as shown by the data obtained with the experiment of RNA protection. The ELISA approach in fact evidences the interaction of antigen/antibody (CAT protein/antiCAT-antibody) only if the antigen is found in the proper and very likely active molecular structure.

## 4. Conclusions

In conclusion, the data discussed in this work evidence that it is possible to define an experimental situation where the cell mortality caused by the exposure to the vesicles is minimal. This is a crucial aspect to maximize the transfection efficiency of catanionic vesicles. The formation of vesicles in the presence of the molecule to be delivered inside the cell is of crucial importance for its internalization within the inner aqueous medium. Furthermore, the vesicles are stable at room temperature and have a relatively long shelf life. They must be stored at room temperature and should not be frozen since this would disrupt their supramolecular structure thus abolishing their capacity of molecular delivery. Catanionic vesicles efficiently deliver genetic material across the cell membrane. Transfection efficiency increases if vesicles are formed in the presence of RNA. Treatment with RNase suggests the internal localization of RNA the nucleic acid. 

## Abbreviations

CAT: Chloramphenicol-acetyltransferase
DLS: Dynamic light scattering
ELISA: Enzyme-linked immunosorbent assay
HEK-293: Human Embryonal Kidney cells
SDS-CTAB: Sodium dodecylsulfate/cetyltrimethylammonium bromide
SDS-DDAB: Didodecyldimethylammonium bromide.
